# A Semi-Continuous Process For Polyphenols Extraction From Sea Buckthorn Leaves

**DOI:** 10.1038/s41598-019-48610-6

**Published:** 2019-08-19

**Authors:** Ioana Asofiei, Ioan Calinescu, Adrian Trifan, Adina Ionuta Gavrila

**Affiliations:** Faculty of Applied Chemistry and Materials Science, Department of Bioresources and Polymer Science, 1-7 Gh. Polizu, Bucharest, Romania

**Keywords:** Chemistry, Green chemistry, Microwave chemistry

## Abstract

Sea buckthorn (*Hippophae Rhamnoides L*.) is an important source of bioactive compounds such as: antioxidants, vitamins, fatty acids, amino acids, and minerals. Sea buckthorn leaves extracts have been proved to have anti-microbial, antioxidant, anti-inflammatory, and anti-viral properties. In this paper, the extraction of polyphenols from sea buckthorn leaves using a semi-continuous small-scale reactor is described. The extraction conditions must not affect the composition and structure of polyphenols. For this reason, the influence of different parameters (residence time, solvent flow rate, stirring rate, reactor type, and solvent pre-heating) on the extraction process were studied. The polyphenolic extracts were analyzed in order to determine the total phenolic content (TPC), the antioxidant capacity and the concentration of the main phenolic compounds. The TPC increases with the stirring rate. Pre-heating the solvent leads to a better yield and reduced residence time. The antioxidant capacity is in concordance with the TPC results. HPLC analysis confirms that the semi-continuous microwave assisted extraction (MAE) does not affect the composition of the extracts. The energy consumption was significantly lower for MAE compared with conventional extraction (CE).

## Introduction

Polyphenols are valuable metabolites synthesized by plants during their growth and they may act as antioxidants, phytoalexins, protective agents against UV light, and attractants for pollinators, among others. The consumption of food rich in these bioactive substances has been accompanied by a reduction in hypertension and endothelial dysfunction, atherosclerosis and dyslipidemia, thrombosis and inflammatory processes related to cardiovascular diseases^1–3^.

Sea buckthorn (*Hippophae Rhamnoides L*.) belongs to *Elaeagnaceae* family and all parts of this plant are rich in a wide range of bioactive substances^4,5^. Sea buckthorn leaves contain valuable bioactive compounds such as tocopherols, carotenoids, flavonoids, sterols, vitamins, tannins, lipids, and minerals. These components are useful in medicinal and pharmaceutical applications due to the antioxidant, anti-inflammatory, antibacterial, antiviral, and antitumor properties^6^.

The extraction of polyphenols from plants is the first step in utilization of these phytochemicals in nutritional supplements, food ingredients, cosmetic and pharmaceutical products. The phenolic compounds can be extracted from dried or fresh plant materials. The most common method to obtain extracts from plants is solvent extraction. The extraction process is influenced by the solvent type, solvent to plant ratio, temperature, extraction time or stirring rate^7,8^. The conventional methods such as Soxhlet extraction or maceration present low efficiency due to long extraction times, high temperatures and polyphenols degradation^9^. In recent years, more efficient extraction processes such as ultrasound and microwave assisted extractions^10–12^, simultaneous ultrasonic-microwave assisted extraction^13,14^, pressurized and supercritical fluid extractions^15–17^, pulsed electric field^18,19^, microwave and enzyme co-assisted extraction^20^ have been developed. The methods used to extract bioactive compounds from plant materials are mostly batch processes. However, Puertolas has evaluated the phenolic extraction during fermentation of red grapes by a continuous pulsed electric field^21^ and Petigny performed a continuous ultrasound assisted extraction of bioactive compounds from boldo leaves^22^.

MAE is an alternative process used for polyphenols extraction from vegetable material, being an environmentally friendly process. This process has many advantages, e.g., shorter extraction time and lower amount of solvent required. An important issue of polyphenols extraction is their thermolability^23^. In order to avoid the polyphenols degradation, the extraction of these heat-sensitive compounds requires a rapid heating and a shorter extraction time. MAE involves volumetric heating and is a controllable process. For this reason, the microwave technique is an attractive alternative for extraction^24,25^. The degradation of sensitive bioactive compounds during extraction can be minimized by using a continuous flow extraction system. Therefore, the residence time of polyphenols in the system is easily controlled by modifying the solvent flow rate, thus reducing the degradation of these thermo-sensitive compounds^26^. The most important advantages of continuous microwave heating are controllable heat distribution, fast increase of temperature, and the possibility of quenching of the products in the flow^27,28^.

The novelty of this study consists in performing the extraction of polyphenols from sea buckthorn leaves using a semi-continuous small-scale reactor. The extraction conditions must not affect the composition and structure of polyphenols. For this reason, the influence of different parameters (residence time, stirring rate, flow rate, and reactor type) on the extraction process was studied. Another aim of this research is to achieve a high concentration of polyphenols in the first fraction collected, thus increasing the efficiency of the process.

## Materials and Methods

### Materials

The sea buckthorn leaves (*Hippophae Rhamnoides L*.) were harvested in the summer of 2017 at Hofigal S.A. in Furculesti. The fresh leaves were dried in an air flow-heating oven at 60 °C to a constant weight. The dried leaves were ground using an electric grinder and screened to a particle size under 0.5 mm. The ground sea buckthorn leaves were dosed in samples of 25 g (in sealed plastic vessels) and stored at 4–5 °C until they were used for the extraction of phenolic compounds.

Folin Ciocalteu reagent (Merck), ethanol and sodium carbonate were of analytical grade. For the HPLC quantification of phenolic compounds, the following standards were used: gallic acid, caffeic acid, chlorogenic acid, catechin, epicatechin, epigallocatechin, ferulic acid, p-coumaric acid, kaempferol-3-O-glucoside, isorhamnetin-3-O-glucoside, rutin, quercetin, kaempferol, and isorhamnetin from Sigma-Aldrich. Trolox (6-hydroxy-2,5,7,8-tetramethy-chroman-2-carboxylic acid), ABTS (2,2-azinobis (3-ethylbenzothiazoline-6-sulfonic acid) diammonium salt), and potassium persulfate were purchased from Sigma-Aldrich.

### Extraction procedure

**Figure 1:**
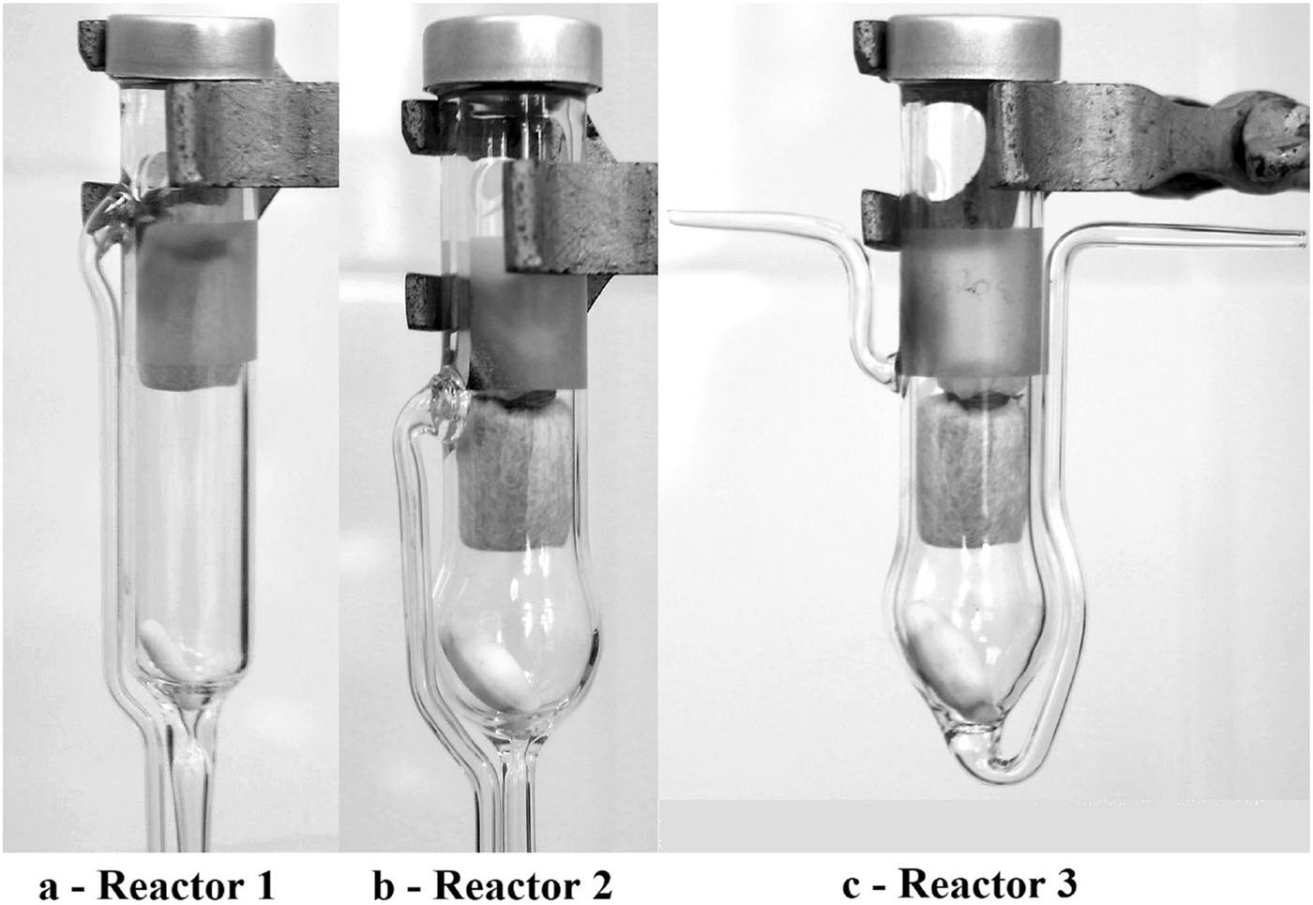
Type of reactor (reactor 1 and 2 are used for MAE and reactor 3 is used for CE).

The semi-continuous MAE of polyphenolic compounds was performed in a microwave applicator (Biotage®Initiator) using a standard reactor modified in order to achieve a semi-continuous process. The experiments of MAE were carried out using two extraction procedures: pre-heating the solvent at a temperature of 60 °C before entering the reactor and introducing the solvent into the reactor at room temperature. The CE of polyphenolic compounds was carried out in a water bath using a heating plate equipped with a temperature controller unit and magnetic stirrer. The experiments were performed using different reactor types presented in Fig. 1 (Fig. 1a,b show the reactors used for semi-continuous MAE, meanwhile Fig. 1c shows the reactor used for semi-continuous CE). In addition, the temperature profile of CE was similar to the one of MAE.

In order to determine the efficiency of semi-continuous extraction, the total amount of polyphenols in sea buckthorn leaves needs to be verified. Thus, a discontinuous multiple extraction was performed using the same extraction conditions as in the semi-continuous process. The procedure of discontinuous multiple extraction consists of adding a fresh portion of solvent over the same plant material after the extract was centrifuged. This approach was repeated until a TPC value above 1.5 mg GAE/g DM was achieved. Thus, after eight cycles the total amount of polyphenols was 216.37 mg GAE/g DM.

The extractions were performed in triplicate using a mixture of 50% ethanol in water, a 20:1 solvent to plant ratio, and were carried out at a temperature of 60 °C using two stirring rates: 300 and 900 rpm. The experiments were conducted considering the following flow rates: 1.5 mL of solvent/min with a residence time of 200 s, 3 mL of solvent/min with a residence time of 100 s and 6 mL of solvent/min with a residence time of 50 s. The mixture was separated using a filter, directly in the reactor. For each experiment eight fractions of supernatant were collected and analyzed fresh every time.

### Determination of total phenolic content

TPC of extracts was determined colorimetrically using the Folin-Ciocalteu method according to International Standard ISO 14502-1 with minor modifications. The fresh extracts were diluted 125 times with distilled water. Further, 0.5 mL of diluted extract was mixed with 5 mL of 10% Folin Ciocalteu reagent and stirred for 5 minutes in order to perform the reaction between them. Next, 1.5 mL of 20% Na_2_CO_3_·10H_2_O and 3 mL of distilled water were added. Before analysis, the samples were kept for 60 min in the dark at room temperature. The absorbance was measured at 760 nm using a Shimadzu UV mini-1240 UV/Visible Scanning Spectrophotometer, 115 VAC. The samples were analyzed in duplicate. The results were quantified as milligrams of gallic acid equivalents per 1 g of dry matter (mg GAE/g DM) using a standard curve corresponding to 1–5 mg/mL gallic acid solution. This analysis method for TPC is also described in our previous work^29^.

### TEAC assay

The antioxidant capacity was performed using 2,2-azino bis-3-ethylbenzthiazoline-sulphonic acid (ABTS) radical scavenging assay and was expressed as Trolox equivalent antioxidant capacity (TEAC). Two aqueous stock solutions containing 7 mM ABTS and 2.45 mM potassium persulfate were prepared. The ABTS radical cation (ABTS•+) resulted by reacting these two solutions (1:1) and keeping the mixture in the dark for 12–16 h (at room temperature). For analysis of polyphenolic extracts, ABTS•+ was diluted with ethanol to an absorbance of 0.70 ± 0.02 at a wavelength of 734 nm. The analysis mixture consists of 1 mL of diluted extract sample, 3 mL of diluted ABTS•+ solution and ethanol up to 5 mL. The absorbance was read after 4 min, with a suitable solvent blank run in each assay. All determinations were performed in duplicate. The percentage inhibition of absorbance at 734 nm was calculated and plotted as a function of antioxidant concentration. The analysis method is also described in our previous work^29^.

### HPLC analysis of major phenolic compounds

The phenolic compounds extracted were further analized by HPLC analysis. The analyses were undertaken using a Jasco HPLC equipped with: UV-2075 detector; PU-2080 plus pump; LG-2080_4 gradient unit; DG-2080_4 degasser; Teknokroma Mediteranea 100 C18 separation column (5 µm, 250 × 0.4 mm ID). Analysis was carried out at a flow rate of 0.5 mL/min using water with 2% v/v acetic acid (solvent A) and methanol (solvent B) under the following gradient program: 0–9 min 70% A and 30% B, 9–18 min 60% A and 40% B, 18–60 min 50% A and 50% B, 60–80 min 100% B and then returned to initial conditions for a 10 min re-equilibration, with total run time of 90 min. The analytes were detected at 270 nm. In order to eliminate the interference of other matrix compounds from polyphenolic aqueous ethanol solutions, they were extracted again using ethyl ether as solvent (50% polyphenols solution and 50% ethyl ether are stirred for 10 min at room temperature). Ethyl ether is evaporated from the extract and the residue is dispersed in 50% aqueous ethanol solution. The polyphenols were identified from these solutions according to retention times and standard addition method. The recovery of polyphenols by extraction in ethyl ether was estimated on solutions of known standards. The extractions and analyses were performed in duplicate. The HPLC method is also described in our previous work^29^.

## Results and Discussion

### Influence of stirring rate and flow rate on the semi-continuous MAE of polyphenols

In order to determine the factors that influence the semi-continuous process of MAE of polyphenols, a series of parameters were studied. Therefore, one of the studied parameters was the influence of the stirring rate on the TPC.

**Figure 2:**
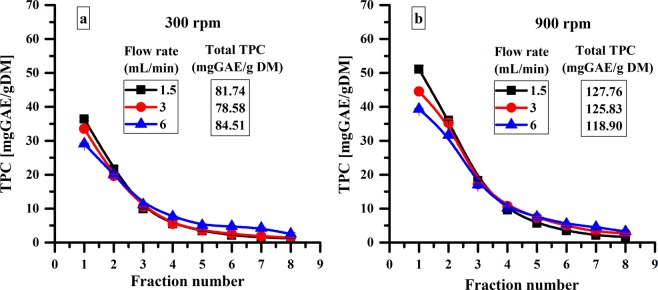
Influence of stirring rate and flow rate on the semi-continuous MAE of polyphenols using reactor 1.

The stirring rate represents an important factor in polyphenols extraction. As shown in Fig. 2, a higher stirring rate leads to an increase in the TPC both in the first fraction and the total amount of polyphenols. Due to an efficient stirring of the extraction medium, a better contact between plant material and solvent is achieved and the TPC increases. In addition, a higher stirring rate causes a mechanical destruction of cell walls facilitating the access of the solvent into the cell which favours the extraction of polyphenols.

The flow rate is another parameter that influences the semi-continuous MAE process. Decreasing the flow rate leads to an important increase of the polyphenolic content in the first fraction (from 39.34 mg GAE/g DM at 6 mL of solvent/min to 51.06 mg GAE/g DM at 1.5 mL of solvent/min) for a stirring rate of 900 rpm (Fig. 2b). Moreover, decreasing the flow rate leads to a higher total amount of polyphenols (from 118.90 mg GAE/g DM at 6 mL of solvent/min to 127.76 mg GAE/g DM at 1.5 mL of solvent/min). This can be explained by a better extraction of polyphenols with the increase of residence time from 50 s to 200 s.

### Influence of reactor type on the semi-continuous MAE of polyphenols

**Figure 3:**
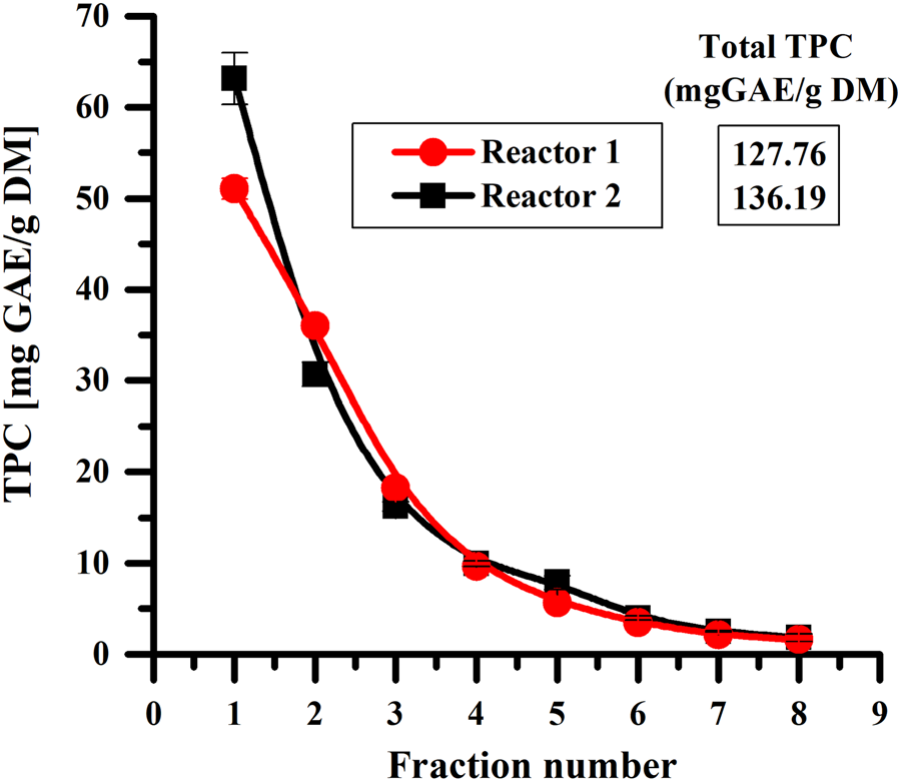
Influence of reactor type on the semi-continuous MAE of polyphenols for a stirring rate of 900 rpm and a flow rate of 1.5 mL/min.

As shown previously, increasing the stirring rate leads to a higher total amount of polyphenols (from 81.74 mg GAE/g DM at 300 rpm to 127.76 mg GAE/g DM at 900 rpm for a flow rate of 1.5 mL/min). An approach to achieve an efficient mixing of the extraction medium is to use a stirrer with large dimensions. Thus, the reactor used for the extractions was modified to allow using a bigger stirrer. The reactors used for semi-continuous MAE of polyphenols from sea buckthorn leaves are shown in Fig. 1a (reactor 1) and 1b (reactor 2). The influence of the reactor type on the semi-continuous MAE of polyphenols was studied at a flow rate of 1.5 mL/min for both reactors as shown in Fig. 3.

Increasing the stirring rate by using a bigger magnetic stirrer leads to a higher TPC in the first fraction (Fig. 3). Although the difference of the TPC values for the other fractions are insignificant, the total amount of polyphenols is higher for reactor 2. This increase of the TPC can be explained by a better mass transfer and by a higher average microwave power for reactor 2 compared with reactor 1.

### Influence of solvent pre-heating on the semi-continuous MAE of polyphenols

**Figure 4:**
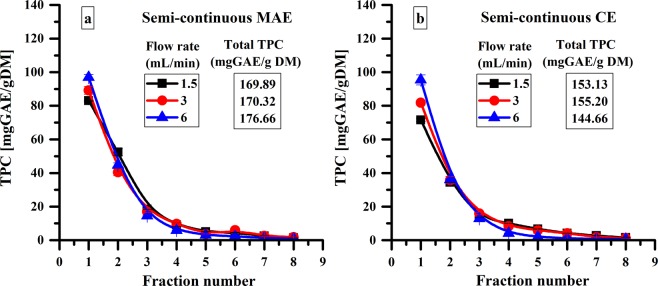
Influence of solvent pre-heating on the semi-continuous MAE and CE of polyphenols using reactor 2 for a stirring rate of 900 rpm.

A strategy to improve the amount of polyphenols in the first fraction for semi-continuous process is the pre-heating of solvent. Before entering the extraction reactor which contains the sea buckthorn leaves, the solvent is heated at 60 °C and then it is allowed to flow continuously through the reactor. The influence of the solvent pre-heating on the TPC of each fraction at different solvent flow rate is presented in Fig. 4.

The solvent pre-heating influences the total amount of polyphenols, increasing the TPC in the first fraction with approximately 25% and the total amount of polyphenols with approximately 20% for a flow rate of 1.5 mL/min (Figs 3 and 4a). Also, the pre-heating of the solvent allows increasing the flow rate from 1.5 mL/min to 6 mL/min without affecting the extraction efficiency. This strategy of solvent pre-heating leads to extracting about 80% of the total amount of polyphenols from sea buckthorne leaves (176.66 mg GAE/g DM for semi-continuous MAE and 216.37 mg GAE/g DM for discontinuous multiple extraction – see Fig. 4a and “Extraction procedure” section).

Semi-continuous conventional extractions were also performed to highlight the influence of microwave assisted process on the polyphenols extraction (Fig. 4b). The experiments were carried out in the same conditions as semi-continuous MAE with solvent pre-heating using the reactor 3 (Fig. 1c).

As shown in Fig. 4a,b, the TPC has a similar behavior during all eight fractions for both extraction methods. Thus, the total amount of polyphenols is higher for semi-continuous MAE compared with semi-continuous CE when only 65–70% of polyphenols are extracted.

### Antioxidant capacity of polyphenolic extracts

**Figure 5:**
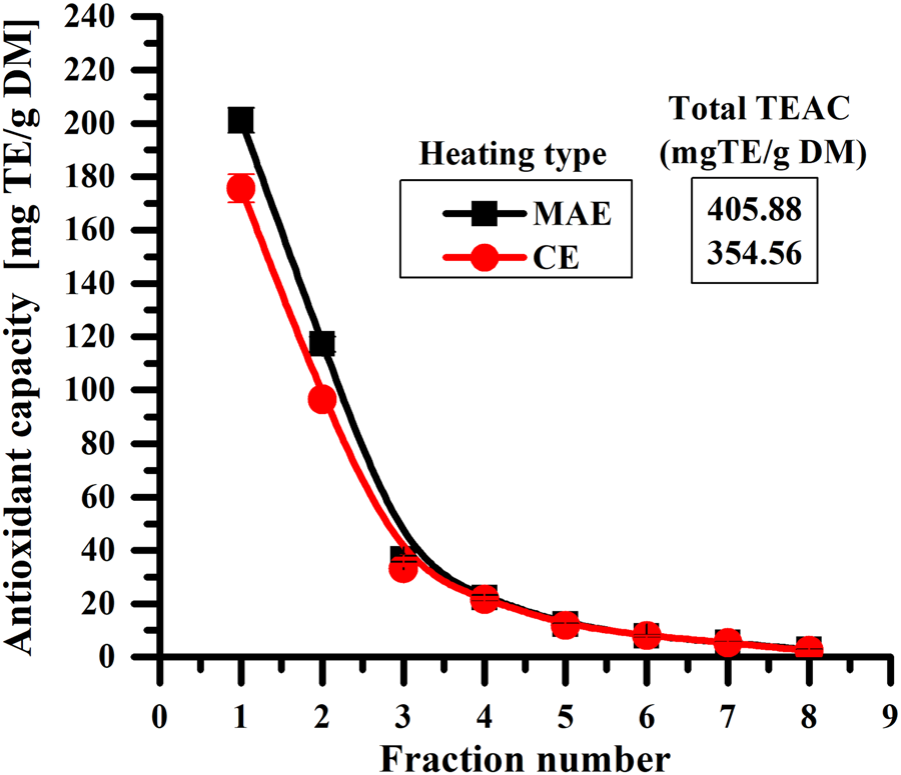
The antioxidant capacity of MAE and CE extracts with solvent pre-heating at a flow rate of 6 mL/min.

The antioxidant capacity was carried out for MAE and CE methods with solvent pre-heating at a flow rate of 6 mL of solvent/min and a stirring rate of 900 rpm. The antioxidant capacity of all collected fractions is in concordance with the TPC values obtained for the same extraction conditions (Figs 4 and 5). The antioxidant capacity is higher for MAE compared with CE for both the first fraction and the total amount of all eight fractions (Fig. 5).

### Polyphenols composition

The quantitative analysis of the main polyphenols for sea buckthorn leaves extracts is presented in Table 1. The HPLC analysis was performed for the first and fourth fractions of the extracts obtained by MAE and CE with solvent pre-heating at a flow rate of 6 mL/min and a stirring rate of 900 rpm. The main components from sea buckthorn leaves extracts for both fractions and methods are gallic acid, catechin, *p*-coumaric and ferulic acids, rutin, kaempferol-3-O-glucoside, isorhamnetin-3-O-glucoside and quercetin. All the identified components are obtained in higher quantities by semi-continuous MAE than by CE. Gallic acid was the predominant polyphenolic species followed by catechin for both analysed fractions.

**Table 1:** Quantitative analysis of the main polyphenols obtained by semi-continuous MAE and CE with solvent pre-heating (flow rate – 6 mL/min, stirring rate – 900 rpm).

Polyphenol compound	Polyphenol compound concentration [mg/g DM]			
	Heating type			
	MAE		CE	
	Fraction 1	Fraction 4	Fraction 1	Fraction 4
Gallic acid	18.02	3.67	12.37	3.42
Catechin	14.62	2.43	9.86	1.62
*p*-Coumaric acid	0.31	0.03	0.28	0.03
Ferulic acid	0.60	0.07	0.52	0.07
Rutin	3.37	0.28	2.69	0.22
Kaempferol-3-*O*-glucoside	2.49	0.19	2.19	0.16
Isorhamnetin-3-*O*-glucoside	2.09	0.27	1.96	0.30
Quercetin	1.64	0.93	1.24	0.88
Total	43.14	7.88	31.11	6.71

As shown in Table 1 the polyphenols compound concentration is much lower in the fourth fraction compared with the first fraction for both methods (semi-continuous MAE and CE). However, the quercetin is extracted harder than the other compounds. A considerable amount of quercetin was extracted in the fourth fraction, which represents 60–70% of the concentration obtained in the first one.

### Energy considerations

During extraction, the microwave power was recorded for all experiments. Further, average microwave power for both extraction time and heating time was calculated. In Table 2 are shown the average microwave power and the amount of energy consumed during MAE and CE for all flow rates used. Pre-heating of the solvent was achieved using a glass heat exchanger, where water was the heating agent for both MAE and CE. Considering the latter, average power and energy were calculated without considering the energy consumption for solvent pre-heating.

**Table 2:** Average power and energy consumption for MAE and CE during extraction.

Flow rate [mL/min]	Average power [W]		Energy [kJ]	
	MAE	CE	MAE	CE
1.5	32.9	600	6.6	120
3	46.5	600	4.6	60
6	54.2	600	2.7	30

As shown in Fig. 4, the total TPC is approximately 15% higher for MAE compared with CE. In addition, we can notice from Table 2 that the energy consumption during the extraction is approximately 15 times lower for MAE compared with CE.

## Conclusions

The aim of this work was to develop an efficient method of MAE of polyphenols from sea buckthorn leaves by a semi-continuous process. To determine the factors that influence this method, different parameters were studied: stirring rate, flow rate, type of reactor and solvent pre-heating. Increasing the stirring rate from 300 rpm to 900 rpm increased the TPC with approximately 40%. Changing the geometry of the reactor to allow using a larger stirrer, that improves the mixing, led to an efficient extraction. Using the modified reactor led to an increase of polyphenolic content with 20% (in the first fraction) compared with the unmodified reactor. The solvent pre-heating strategy improved the total amount of extracted polyphenols reaching 80% of the amount of phenolic compounds found in sea buckthorn leaves. Also, pre-heating of the solvent allowed decreasing the residence time to 50 s without affecting the extraction efficiency. The antioxidant capacity was in concordance with the TPC values. The chemical composition of sea buckthorn leaves extracts is not affected by MAE. Quercetin was the only compound with a high concentration in the fourth fraction for both methods. The energy consumption was approximately 15 times lower for MAE compared with CE. In conclusion, the optimal conditions for semi-continuous process of MAE of polyphenols from sea buckthorn leaves using a modified reactor were a stirring rate of 900 rpm and a flow rate of 6 mL/min with solvent pre-heating.
